# HEARTS in the Americas clinical pathway. Strengthening the decision support system to improve hypertension and cardiovascular disease risk management in primary care settings

**DOI:** 10.3389/fcvm.2023.1102482

**Published:** 2023-04-26

**Authors:** Andres Rosende, Donald J. DiPette, Ramon Martinez, Jeffrey W. Brettler, Gonzalo Rodriguez, Eric Zuniga, Pedro Ordunez

**Affiliations:** ^1^ Department of Non-Communicable Diseases and Mental Health, Pan American Health Organization, Washington, DC, United States; ^2^ School of Medicine Columbia, University of South Carolina, Columbia, SC, United States; ^3^ Department of Health Systems Science, Kaiser Permanente Bernard J. Tyson School of Medicine, Pasadena, CA, United States; ^4^ Southern California Permanente Medical Group, Los Angeles, CA, United States; ^5^ Consultant for HEARTS in the Americas, PAHO/WHO Office in Argentina, Buenos Aires, Argentina; ^6^ Antofagasta Health Service, University of Antofagasta, Antofagasta, Chile

**Keywords:** cardiovascular diseases, hypertension, clinical protocols, critical pathways, public health, quality improvement, implementation science

## Abstract

**Background:**

HEARTS in the Americas is the regional adaptation of the WHO Global HEARTS Initiative. It is implemented in 24 countries and over 2,000 primary healthcare facilities. This paper describes the results of a multicomponent, stepwise, quality improvement intervention designed by the HEARTS in the Americas to support advances in hypertension treatment protocols and evolution towards the Clinical Pathway.

**Methods:**

The quality improvement intervention comprised: 1) the use of the appraisal checklist to evaluate the current hypertension treatment protocols, 2) a peer-to-peer review and consensus process to resolve discrepancies, 3) a proposal of a clinical pathway to be considered by the countries, and 4) a process of review, adopt/adapt, consensus and approval of the clinical pathway by the national HEARTS protocol committee. A year later, 16 participants countries (10 and 6 from each cohort, respectively) were included in a second evaluation using the HEARTS appraisal checklist. We used the median and interquartile scores range and the percentages of the maximum possible total score for each domain as a performance measure to compare the results pre and post-intervention.

**Results:**

Among the eleven protocols from the ten countries in the first cohort, the baseline assessment achieved a median overall score of 22 points (ICR 18 −23.5; 65% yield). After the intervention, the overall score reached a median of 31.5 (ICR 28.5 −31.5; 93% yield). The second cohort of countries developed seven new clinical pathways with a median score of 31.5 (ICR 31.5 −32.5; 93% yield). The intervention was effective in three domains: 1. implementation (clinical follow-up intervals, frequency of drug refills, routine repeat blood pressure measurement when the first reading is off-target, and a straightforward course of action). 2. treatment (grouping all medications in a single daily intake and using a combination of two antihypertensive medications for all patients in the first treatment step upon the initial diagnosis of hypertension) and 3. management of cardiovascular risk (lower BP thresholds and targets based on CVD risk level, and the use of aspirin and statins in high-risk patients).

**Conclusion:**

This study confirms that this intervention was feasible, acceptable, and instrumental in achieving progress in all countries and all three domains of improvement: implementation, blood pressure treatment, and cardiovascular risk management. It also highlights the challenges that prevent a more rapid expansion of HEARTS in the Americas and confirms that the main barriers are in the organization of health services: drug titration by non-physician health workers, the lack of long-acting antihypertensive medications, lack of availability of fixed-doses combination in a single pill and cannot use high-intensity statins in patients with established cardiovascular diseases. Adopting and implementing the HEARTS Clinical Pathway can improve the efficiency and effectiveness of hypertension and cardiovascular disease risk management programs.

## Introduction

Cardiovascular disease (CVD), mainly ischemic heart disease (IHD) and stroke, causes over 2 million deaths annually in the Americas and has an enormous negative socioeconomic impact (1). Hypertension, the main modifiable risk factor for CVD, affects more than one-third of adults in this region. However, although its treatment is very cost-effective, available, affordable, and safe, only 32.3% of men and 40.9% of women in the Americas have hypertension controlled (blood pressure <140/90 mmHg) (2, 3). Additionally, less than 30% of people with known CVD are treated with evidence-based, proven medications (blood pressure-lowering medications, statins, and aspirin) for secondary prevention (4), underscoring the health system's shortcomings. Indeed, if the Americas improves population-based hypertension control from the current level of 36% to a target of 50%, an estimated 419,924 CVD deaths could be averted (3). Furthermore, if secondary CVD prevention were expanded, many more deaths could be averted.

To address these challenges, the Pan American Health Organization (PAHO) initiated HEARTS in the Americas (5). It is being implemented in 24 countries and over 2,000 primary health care (PHC) facilities. It is a program poised to become the institutionalized model of care for hypertension and CVD risk management in PHC settings by 2025. One of its systematic interventions is implementing a standardized and directive hypertension treatment protocol (6). As a result, most HEARTS countries are moving from hypertension guidelines—without protocols—to standardized treatment protocols based on the best pharmacological options available and affordable in each country. HEARTS protocols emphasize using two antihypertensive medicines from complementary pharmacologic classes in separate pills to initiate treatment upon the diagnosis of hypertension (7). In addition, several countries are taking effective steps to implement a preferred protocol based on a fixed-dose combination (FDC) of antihypertensive drugs in a single pill (8).

At the end of 2021, the WHO released the Guideline for the Pharmacological Treatment of Hypertension in Adults (9). In parallel, HEARTS in the Americas delineated the key drivers for hypertension control (10), a set of specific recommendations to improve the clinical and managerial processes in the PHC setting. Based on these developments, HEARTS in the Americas created a methodology to incorporate these new recommendations resulting in the HEARTS Clinical Pathway for Hypertension and CVD Risk Management, which should help countries update and shape their treatment protocols (11).

This paper aims to describe the main results of a multicomponent, stepwise, and quality improvement intervention designed by the HEARTS in the Americas to support advances in hypertension treatment protocols and evolution towards the Clinical Pathway in countries implementing HEARTS. This intervention is expected to identify improvement areas, reveal the main challenges, and extend best practices through a more standardized and comprehensive approach to hypertension and CVD risk management in PHC. As far as we know, it is the first time that a process of this nature has been carried out and reached so many countries simultaneously. This intervention can serve as an example to other regions of the world to move towards a new clinical and managerial paradigm in hypertension control programs globally.

## Materials and methods

### Intervention design

HEARTS in the Americas designed a multicomponent, stepwise, and quality improvement intervention. It comprises the following steps: (1) the use of the appraisal checklist to evaluate the current hypertension treatment protocols by external and national experts, (2) a peer-to-peer review and consensus process to resolve discrepancies among external and national experts, (3) a proposal of a clinical pathway to be considered by the countries, and (4) a process of review, adopt/adapt, consensus and approval of the clinical pathway by the national HEARTS protocol committee.

**Figure 1:**
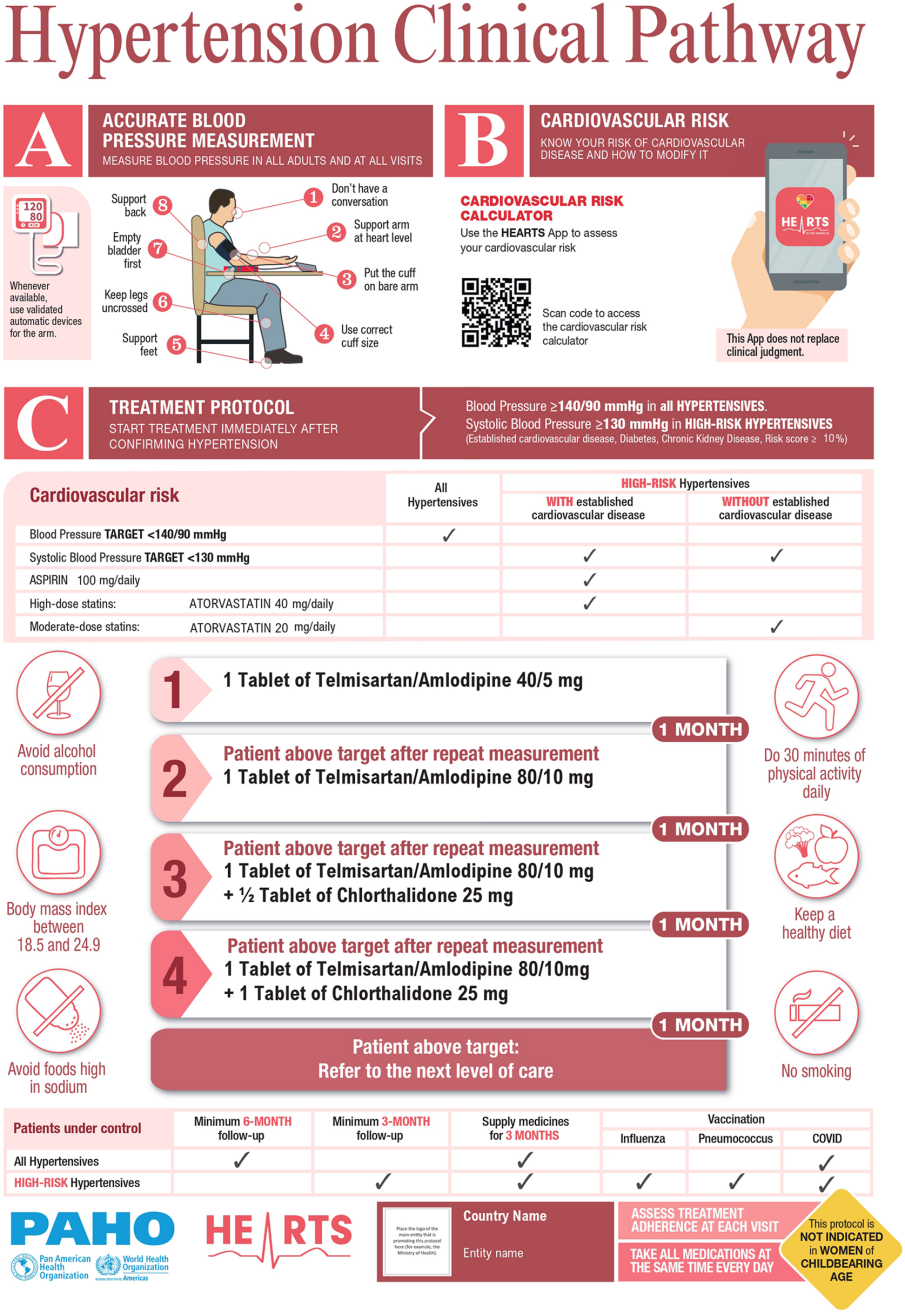
HEARTS in the americas hypertension clinical pathway*. *The medications serve as examples and can be replaced with any two medications from any of the three drug classes (ACEis/ARBs, CCBs or thiazide/thiazide-like diuretics). Start with a single-pill combination (fixed-dose combination) or two individual pills if FDC is not available. Figure was prepared by authors. See Ref (11).

HEARTS in the Americas appraisal checklist and its Clinical Pathway (Figure 1) has been published previously (12). Briefly, HEARTS in the Americas established a core advisory group from high and middle-income countries with proven clinical experience in hypertension management (internal medicine, cardiology, nephrology, and public health) and in-depth knowledge of the HEARTS model. The advisory group defined the attributes and components of a preferred treatment protocol, created the appraisal checklist, and delineated the HEARTS Clinical Pathway. The appraisal checklist and the Clinical Pathway were based on the recommendations from the treatment protocol model included in the WHO HEARTS technical package (13), the HEARTS in the Americas specific recommendations to improve treatment protocols (7), the 2021 WHO hypertension guideline (9), and the HEARTS in the Americas key drivers for hypertension control (10).

The HEARTS appraisal checklist is comprised of 34 questions, organized into three domains: (1) requirements to optimize the implementation of a hypertension treatment protocol, (2) blood pressure pharmacologic treatment, and (3) CVD risk management (see Supplementary Table S1). All these questions were weighted equally, giving 1 point if the answer was positive, 0 points if negative, and 0.5 points if partial. Therefore, the maximum possible score is 34, composed of the sum of the partial scores of each domain: 15, 10, and 9 possible points, respectively.

### Baseline evaluation and intervention

By August 2021, 12 countries that had developed a hypertension treatment protocol were invited to participate in the improvement process. Ten of twelve countries (Argentina, Chile, Cuba, Ecuador, Dominican Republic, Mexico, Peru, Panama, St. Lucia, and Trinidad & Tobago) agreed to participate. In addition, Mexico contributed with two local protocols, one for the State of Chiapas and one for Sonora. As a result, ten countries were included to receive the intervention (first cohort).

First, external experts, using the appraisal checklist, evaluated these 11 protocols. In parallel, experts from each country used the same checklist and did the same process to identify areas for improvement. Then, separate peer-to-peer meetings were held for each country to compare both evaluations, discuss discrepancies, and reach a consensus on the final score. This evaluation resulted in a baseline overall quality score for each protocol. Then, based on the assessment and using the HEARTS Clinical Pathway as a standard, participants' countries committed to initiating a process to adjust their treatment protocols and move toward the clinical pathway standard format.

A second cohort of eight new countries (Bahamas, Bolivia, Brazil, British Virgin Islands, Costa Rica, Dominica, El Salvador, and Guyana), and the state of Yucatan in Mexico, joined HEARTS after evaluation of the protocols had been completed from the first cohort of countries. The second cohort of countries, with no treatment protocols, was trained to use the appraisal checklist to guide the development of their first hypertension protocol under the HEARTS Pathway format.

### Post-intervention evaluation

A year later, 16 participants countries (10 and 6 from each cohort, respectively) were included in a second evaluation using the HEARTS appraisal checklist. Fourteen countries defined their clinical pathways, while Argentina and Panama continued to use their previous hypertension treatment protocols. We compared pre and post interventions scores for the first cohort. For the second cohort, it was used in the post-intervention score. The results were aggregated by domain and broken down by country to identify specific areas for improvement and challenges.

### Statistical analysis

**Figure 2:**
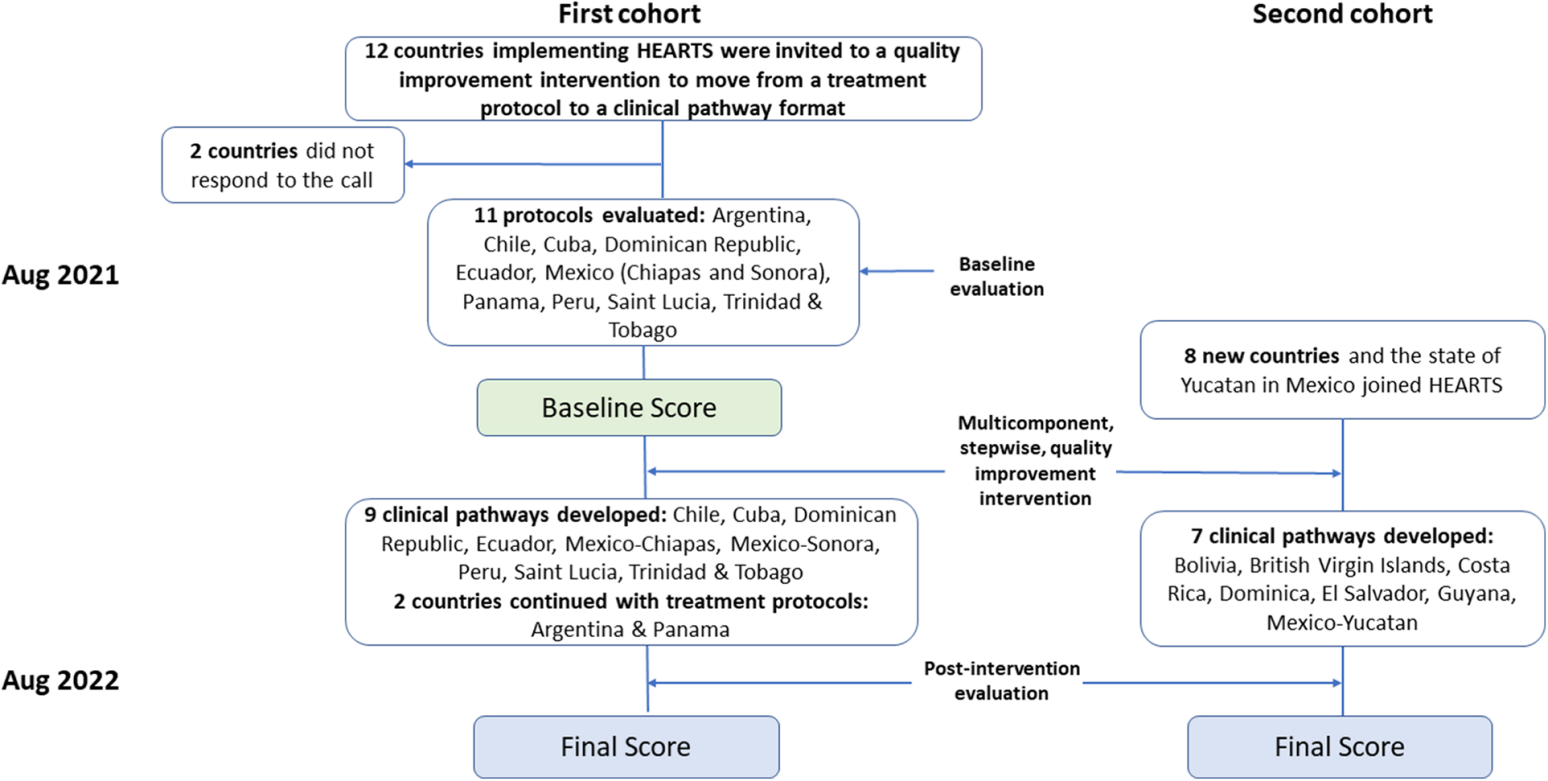
HEARTS in the americas. Intervention and evaluation process to move from a standardized hypertension treatment protocol to a CVD risk management clinical pathway.

To compare the results pre and post-intervention, we used two metrics: (a) the median and interquartile scores range, and (b) the percentages of the maximum possible total score for each domain as a performance measure (Flowchart, Figure 2). Statistical analyses were performed using a standard software package (Stata, version 13.0; StataCorp).

## Results

### Overall improvement

Among the eleven protocols from the ten countries of the first cohort, the baseline assessment achieved a median overall score of 22 points (ICR 18–23.5; performance of 65%). After the intervention, the overall score reached a median of 31.5 (ICR 28.5–31.5; performance of 93%). Thus, eight countries moved towards the clinical pathway. Argentina and Panama kept using their previous protocols and made only minor changes to those.

**Figure 3:**
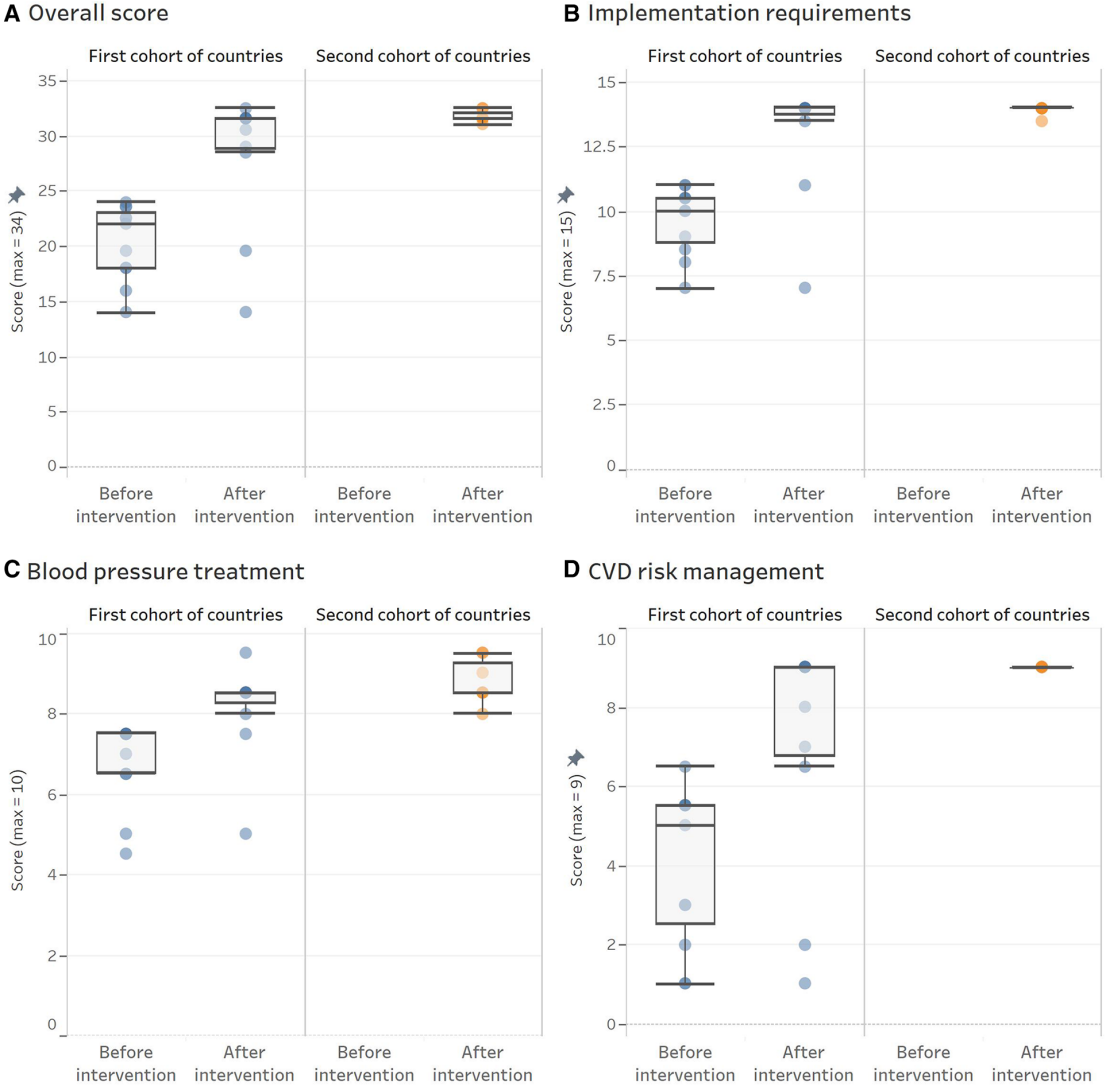
HEARTS appraisal checklist overall and domain-specific scores obtained before and after the intervention by cohort of countries. (**A**) Overall score. (**B**) Implemenation requirements. (**C**) Blood pressure treatment. (**D**) CVD risk management. Each dot represents an evaluated protocol. The overimposed boxplots provide information about the central trend and variation before and after the intervention by cohort of countries. Panel **A** shows overall scores, and panel **B**, **C**, and **D** presents scores for each domain.

The second cohort of countries assisted by the same expert group, developed seven new clinical pathways (Bolivia, British Virgin Islands, Costa Rica, Dominica, El Salvador, Guyana, and Mexico-Yucatan), with a median score of 31.5 (ICR 31.5–32.5; performance of 93%), comparable to that achieved by the countries from the first cohort. A detailed description of these results is shown in Figures 3 (by domains) and 4 (by countries) and described below (for additional information, see Supplementary Tables S2, S3).

**Figure 4:**
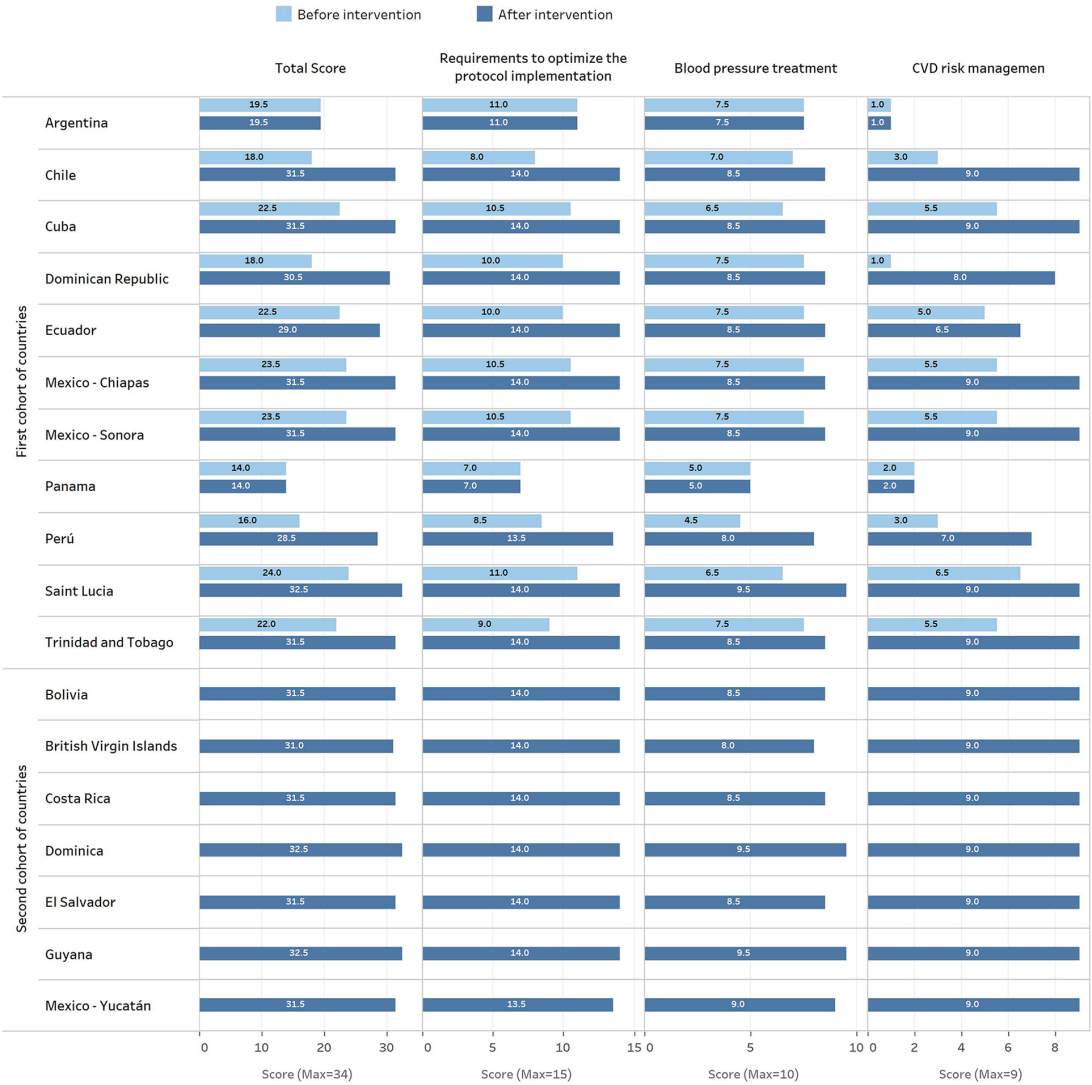
HEARTS appraisal check list scores before and after the evaluation and improvement process by county-protocol.

### Requirements to optimize the protocol implementation in PHC settings

Under the guiding principle that a clinical pathway must be feasible to implement, the objective was to evaluate and modify its structural aspects to facilitate the program's implementation in a PHC setting. At baseline, protocols of the first cohort reached a median score of 10 (ICR 8.5–10.5; performance of 67%). However, after the intervention, the score increased to a median of 14 (ICR 13.5–14; performance of 93%). This result is due to improvements in the recommendations on clinical follow-up intervals, frequency of medication refills, systematic repetition of blood pressure measurement when the first reading is out of the target, and a more straightforward course of action. In contrast, no country made progress in allowing non-physician health workers, trained and under supervision, to manage antihypertensive treatment while following the approved protocol.

The second cohort defined their clinical pathways, achieving a median score of 14 (ICR 14–14; performance of 93%). This performance was similar to that of the countries in the first cohort after the intervention. Likewise, its main gap continues to be the lack of regulations allowing antihypertensive treatment management by non-physician health workers.

### Blood pressure pharmacologic treatment

This domain includes all items related to hypertension pharmacologic treatment, such as the classes of antihypertensive medication and individual medications within each class, medication doses, and how to use them. At baseline, the first cohort achieved a median score of 7.5 (ICR 6.5–7.5; performance of 75%). However, after the improvement process, the performance increased to a median score of 8.5 (ICR 8–8.5; performance 85%). The main improvements were observed in the recommendation of grouping all medications in a single daily intake and using a combination of two antihypertensive medications for all patients in the first treatment step upon the initial diagnosis of hypertension. However, major gaps persist in the availability of long-acting medications and FDC.

The clinical pathways of the countries included in the second cohort reached a median score for this domain of 8.5 (ICR 8.5–9.5; performance of 85%), similar to that achieved for the countries from the first cohort after the intervention. However, the significant gaps are the same, the lack of availability of long-acting medication and FDC.

In summary, after the intervention, all countries selected medications corresponding to the three first-line pharmacological groups recommended by most, if not all guidelines, including the WHO: angiotensin-converting enzyme inhibitors (ACEIs) or angiotensin receptor blockers (ARBs), dihydropyridine calcium channel blockers (CCBs), and thiazide/thiazide-like agents (TZ-TZL). All countries selected amlodipine as the CCB. Most selected hydrochlorothiazide, except three countries that chose the longer-acting TZL agent, chlorthalidone, and one country chose the TZL agent, indapamide. All countries, except three, started treatment by combining two drugs in separate pills, mainly an ACEI or an ARB, with a CCB or TZ-TZL. Only Saint Lucia, Dominica, and Guyana used an FDC pill (Table 1).

**Table 1:** Medications included in clinical pathways and protocols of the HEARTS countries.

Clinical Pathways and other Treatment Protocols							
HEARTS Countries	Hypertension Treatment Protocol					Concomitant treatment in high-risk patients	
	Step 1	Step 2	Step 3	Step 4	Step 5	Primary prevention	Secondary prevention
Argentina	Amlodipine 5 mg	Amlodipine 5 mg Losartan 50 mg	Amlodipine 5 mg Losartan 100 mg	Amlodipine 10 mg Losartan 100 mg	Amlodipina 10 mg Losartan 100 mg HCTZ 25 mg	No specific recommendation	No specific recommendation
Bolivia	Losartan 50 mg HCTZ 25 mg	Losartan 100 mg HCTZ 50 mg	Losartan 100 mg HCTZ 50 mg Amlodipine 5 mg	Losartan 100 mg HCTZ 50 mg Amlodipine 10 mg		Atorvastatin 20 mg	Atorvastatin 40 mg Aspirin 100 mg
British Virgin Islands	Amlodipine 10 mg	Amlodipine 10 mg Lisinopril 20 mg	Amlodipine 10 mg Lisinopril 40 mg	Amlodipine 10 mg Lisinopril 40 mg Indapamide 1.5 mg		Atorvastatin 20 mg	Atorvastatin 40 mg Aspirin 81 mg
Chile	Losartan 50 mg Amlodipine 5 mg	Losartan 100 mg Amlodipine 10 mg	Losartan 100 mg Amlodipine 10 mg HCTZ 25 mg	Losartan 100 mg Amlodipine 10 mg HCTZ 50 mg		Atorvastatin 20 mg	Atorvastatin 40–80 mg Aspirin 100 mg
Costa Rica^#^	Enalapril 20 mg Amlodipine 5 mg	Enalapril 40 mg Amlodipine 10 mg	Enalapril 40 mg Amlodipine 10 mg HCTZ 25 mg	Enalapril 40 mg Amlodipine 10 mg HCTZ 50 mg		Lovastatin 40–80 mg	Rosuvastatin 20–40 mg Aspirin 100 mg
Cuba^#^	Enalapril 20 mg HCTZ 12.5 mg	Enalapril 40 mg HCTZ 25 mg	Enalapril 40 mg HCTZ 25 mg Amlodipine 5 mg	Enalapril 40 mg HCTZ 25 mg Amlodipine 10 mg		Atorvastatin 20 mg	Atorvastatin 40 mg Aspirin 100 mg
Dominica^*^	Lisinopril 20 mg HCTZ 12.5 mg	Lisinopril 40 mg HCTZ 25 mg	Lisinopril 40 mg HCTZ 25 mg Amlodipine 5 mg	Lisinopril 40 mg HCTZ 25 mg Amlodipine 10 mg		Atorvastatin 20 mg	Atorvastatin 40 mg Aspirin 100 mg
Dominican Republic	Candesartan 16 mg Amlodipine 5 mg	Candesartan 32 mg Amlodipine 10 mg	Candesartan 32 mg Amlodipine 10 mg HCTZ 25 mg	Candesartan 32 mg Amlodipine 10 mg HCTZ 25 mg		Simvastatin 20 mg	Simvastatin 40 mg Aspirin 100 mg
Ecuador	Losartan 100 mg Amlodipine 5 mg	Losartan 100 mg Amlodipine 5 mg Chlorthalidone 25 mg	Losartan 100 mg Amlodipine 5 mg Chlorthalidone 50 mg	Losartan 100 mg Amlodipine 10 mg Chlorthalidone 50 mg		Simvastatin 20 mg	Simvastatin 40 mg Aspirin 100 mg
El Salvador^#^	Enalapril 20 mg Amlodipine 5 mg	Enalapril 40 mg Amlodipine 10 mg	Enalapril 40 mg Amlodipine 10 mg HCTZ 25 mg	Enalapril 40 mg Amlodipine 10 mg HCTZ 50 mg		Atorvastatin 20 mg	Atorvastatin 40 mg Aspirin 100 mg
Guyana^*^	Ramipril 5 mg Amlodipine 5 mg	Ramipril 10 mg Amlodipine 10 mg	Ramipril 10 mg Amlodipine 10 mg HCTZ 25 mg	Ramipril 10 mg Amlodipine 10 mg HCTZ 50 mg		Atorvastatin 20 mg	Atorvastatin 40 mg Aspirin 100 mg
Mexico–Chiapas	Telmisartan 40 mg Amlodipine 5 mg	Telmisartan 80 mg Amlodipine 10 mg	Telmisartan 80 mg Amlodipine 10 mg HCTZ 12.5 mg	Telmisartan 80 mg Amlodipine 10 mg HCTZ 25 mg		Atorvastatin 20 mg	Atorvastatin 40 mg Aspirin 100 mg
Mexico–Sonora	Telmisartan 40 mg Amlodipine 5 mg	Telmisartan 80 mg Amlodipine 10 mg	Telmisartan 80 mg Amlodipine 10 mg HCTZ 12.5 mg	Telmisartan 80 mg Amlodipine 10 mg HCTZ 25 mg		Atorvastatin 20 mg	Atorvastatin 40 mg Aspirin 100 mg
Mexico–Yucatan	Telmisartan 40 mg Amlodipine 5 mg	Telmisartan 80 mg Amlodipine 10 mg	Telmisartan 80 mg Amlodipine 10 mg Chlorthalidone 25 mg			Atorvastatin 20 mg	Atorvastatin 40 mg Aspirin 100 mg
Panama	ACEi or ARB or CCB or TZ at 50% of maximum dose	1 drug at maximum dose or 2 drugs at 50% of maximum dose	2 drugs at maximum dose or 3 drugs at 50% of maximum dose	3 drugs at maximum dose	3 drugs at maximum dose + Spironolactone 12.5 to 25 mg	No specific recommendation	No specific recommendation
Peru^‡^	Losartan 100 mg HCTZ 12.5 mg	Losartan 100 mg HCTZ 12.5 mg Amlodipine 5 mg				No specific recommendation	Atorvastatin 40 mg
Sanit Lucia^*^	Losartan 50 mg Amlodipine 5 mg	Losartan 100 mg Amlodipine 10 mg	Losartan 100 mg Amlodipine 10 mg Chlorthalidone 12.5 mg	Losartan 100 mg Amlodipine 10 mg Chlorthalidone 25 mg		Atorvastatin 20 mg	Atorvastatin 40 mg Aspirin 81 mg
Trinidad & Tobago	Lisinopril 10 mg Amlodipine 5 mg	Lisinopril 20 mg Amlodipine 5 mg	Lisinopril 20 mg Amlodipine 10 mg	Lisinopril 40 mg Amlodipine 10 mg Bendrofluazide 2.5 mg		Rosuvastatin 20 mg	Rosuvastatin 40 mg Aspirin 81 mg

ARB, angiotensin receptor blocker; ACEi, angiotensin-converting enzyme inhibitor; CCB, calcium channel blocker; HCTZ, hydrochlorothiazide; TZ, thiazide/thiazide like agent.

^*^ Uses a single-pill combination of the first two drugs.

^#^ Enalapril is used twice a day.

^‡^ Uses Losartan twice a day and recommends Enalapril as second option.

### Cardiovascular disease risk management

This domain comprises the CVD risk evaluation, BP thresholds and targets based on CVD risk level, and complementary therapy with aspirin and statins, when appropriate. It showed a greater improvement among the three domains evaluated. Protocols of the first cohort went from a median score of 5 (ICR 2–5.5; performance of 55%) to a median score of 9 (ICR 6.5–9; performance of 100%). The main improvements were in the recommendation of lower BP thresholds and targets in high-risk patients and the recommendation to use aspirin and statins in this population group. However, some countries do not select a high-intensity statin recommended for treating patients with established CVD. For instance, Argentina and Panama did not include treatment with statins; Peru did not include aspirin and only recommended statins among patients with established CVD. In addition, Dominican Republic and Ecuador, despite including statins in their clinical pathways, did not recommend high-intensity therapy in secondary prevention because both have only simvastatin (Table 1). Among the second cohort of countries, the performance of this domain was perfect for all of them, reaching a score of 9.

One of the most innovative additions to the HEARTS Clinical Pathway was the introduction of vaccination against influenza, pneumococcus, and COVID-19. The vast majority of countries incorporated these recommendations.

## Discussion

HEARTS in the Americas has advanced across the region due to the leadership of the Ministries of Health, the growing support of professional organizations and civil society, and the generosity of partners and donors (14–16). Likewise, the HEARTS' successful implementation strategy, the innovative and practical solutions to catalyze health system changes, and the application of a set of guiding principles, all co-created by the participating countries and PAHO, have been relevant (6, 17–19).

Indeed, countries actively participated in the improvement process because HEARTS in the Americas is a community of practice with a shared vision and common goals. Moreover, most countries moved forward in parallel, resulting in clinical pathways with high consistency and minimal clinical variability, because a consensus methodology and standardized checklist were used. Furthermore, the HEARTS Clinical Pathway prototype played a key role in shaping clinical pathways in participating countries. Finally, given that each country developed a process of internal consensus adjusted to local conditions, it is expected that the clinical pathway adopted will progressively become, in addition to a normative document, a widely used and accepted clinical tool feasible to implement in the PHC settings (5, 6, 17).

This study also highlights the challenges that prevent a more rapid expansion of HEARTS in the Americas and confirms that the main barriers are in the organization of health services. For instance, as proof that the system is not fully ready for more innovative changes, and although there is much evidence in its favor (10, 20, 21), drug titration by non-physician health workers, such as nurses and pharmacists, even under the supervision and guidance of an approved treatment protocol, remains a significant issue. Although this topic requires further study, traditions, culture, and normative elements seem to coexist and emerge as barriers that prevent the construction of a more effective and efficient system.

The HEARTS Clinical Pathway reflects the recommendations of the WHO and the world's best-known hypertension guidelines (9, 22). Indeed, a key qualitative advance in this process has been that most countries have decided to initiate pharmacologic treatment by combining two pills of different, complementary classes in the initial treatment step of the patient with newly diagnosed hypertension. However, a significant barrier to the HEARTS Clinical Pathway is the continued existence of outdated medication formularies. For instance, most countries do not yet include medications that have all of the characteristics of an ideal medication for the treatment of hypertension (8) but instead use the best their national medicine formularies can ensure. Thus, three countries continue to use enalapril, and six continue to use losartan. Indeed, enalapril should be administered twice daily, while losartan, although it can be taken once daily, is the ARB with the shortest half-life (8). Consequently, the lack of long-acting antihypertensive medications and the lack of availability of FDCs in a single pill are significant barriers to achieving a more effective protocol. Indeed, despite the compelling benefits of using FDCs, such as achieving more rapid blood pressure control with significantly greater adherence and persistence to care, neither the countries nor the PAHO's Strategic Fund, an effective pooled procurement mechanism, have yet to obtain competitive prices that allow access to these medicines (23).

Implementing a standardized, straightforward, simple, and directive pharmacologic treatment protocol was a significant step forward in the clinical management of hypertension in the first HEARTS countries. However, a recurring concern from implementing countries is that the treatment protocol seemed too top-down driven and focused primarily on hypertension (11). Certainly, hypertension and diabetes have overlapping risk factors that lead to common pathways of complications and target organ damage. For example, elevated glucose and blood pressure accelerate atherosclerosis, endothelial dysfunction, and vascular injury (24). In addition, these mechanisms give rise to macrovascular (IHD, stroke, aortic disease, and peripheral arterial disease) and microvascular disease (chronic kidney disease, neuropathy, and retinopathy) (25). Therefore, the HEARTS clinical pathway broadens the scope of care and promotes a more integrated approach, including hypertension, diabetes, chronic kidney disease, and secondary CVD prevention in patients with established CVD (26). However, to maximize this opportunity, countries need better access to medications. One key example is the ability to use high-intensity statins in patients with established CVD.

The HEARTS clinical pathway goes further to show its integrative and contemporary essence. For example, despite a well-known association between respiratory infectious diseases and cardiovascular complications (27–29), the vaccination rate among patients at high risk for CVD is very low (30). Therefore, the HEARTS Clinical Pathway incorporated an immunization chart as part of the continuum of care. These recommendations increase the integration between communicable and non-communicable diseases, reinforcing the perspective of vaccination as a preventive strategy for CVD and preparation for future pandemics (31–33). This effort will effectively address the CVD burden, strengthen the resilience of health systems, and defend against the current COVID-19 pandemic and future public health emergencies (34).

### Limitations

This approach has important limitations. Notably, the HEARTS clinical pathway does not work in a vacuum or as a stand-alone intervention. On the contrary, it operates as a critical element in a complex health system intervention such as HEARTS. Indeed, any health system intervention to be successful and sustainable requires bold leadership, a skilled and engaged workforce, and a process of learning, acceptance, financially secure, incentives, and continuous quality improvement. Therefore, its sustainability will have to stand the test of time and depends on the health system's maturity and the soundness of the strategies adopted in each context.

### Future directions

Adopting and implementing the HEARTS Clinical Pathway in PHC settings can simplify and integrate hypertension management and secondary CVD prevention, improving the efficiency and effectiveness of hypertension programs while optimizing the pharmaceutical market and supply chain (better and more affordable medicines). In addition, a high-quality, standardized clinical pathway in the context of universal access to healthcare can help address inequalities and disparities in health service delivery by ensuring the best standards for CVD prevention and treatment, regardless of economic and social differences. The institutionalization of the HEARTS clinical pathway should be the next step in the right direction.

Although there is still a long journey ahead between having a good implementation tool -a clinical pathway- and achieving outstanding population control for hypertension, the development and adoption of the clinical pathway by all implementing countries is a milestone in the implementation of HEARTS in the Americas in the path to reduce the burden of the deadliest disease of the contemporary era.

## Conclusions

In summary, this study confirms that this quality improvement intervention conducted by HEARTS in the Americas was feasible, acceptable, and instrumental in quickly adopting the new WHO hypertension guideline recommendations and HEARTS key drivers for hypertension control. Almost all countries in the first cohort progressed toward a high-quality clinical pathway. Moreover, the newly implementing countries, including in the second cohort, reached this milestone faster and with less variability. Indeed, progress in all countries and all three domains of the clinical pathway (implementation, blood pressure treatment, and CVD risk management) under evaluation were apparent.

## Data Availability

The original contributions presented in the study are included in the article/**Supplementary Materials**, further inquiries can be directed to the corresponding author/s.
